# Ripening of Pomegranate Skin as Revealed by Developmental Transcriptomics

**DOI:** 10.3390/cells11142215

**Published:** 2022-07-16

**Authors:** Idit Ginzberg, Adi Faigenboim

**Affiliations:** Institute of Plant Sciences, Agricultural Research Organization, Volcani Institute, 68 HaMacabim Road, P.O. Box 15159, Rishon LeZion 7505101, Israel; adif@volcani.agri.gov.il

**Keywords:** anthocyanin, fruit peel, hydrolyzable tannin, non-climacteric fruit, plant growth regulator, *Punica granatum*

## Abstract

The appearance of pomegranate (*Punica granatum* L.) fruit is highly important for its marketing. The primary concerns are obtaining sufficient red pigment accumulation and minimal cracking of the fruit skin (the outer red layer of the peel). We analyzed the skin transcriptome of pomegranate cv. Wonderful at distinct time points of fruit development to characterize the processes that occur in the skin during fruit ripening and which may reflect on processes in the whole fruit, such as the non-climacteric nature of pomegranate. The data suggested a ripening mechanism in pomegranate skin that differs from that in strawberry—the model plant for non-climacteric fruit where abscisic acid is the growth regulator that drives ripening—involving ethylene, polyamine, and jasmonic acid pathways. The biosynthetic pathways of important metabolites in pomegranate—hydrolyzable tannins and anthocyanins—were co-upregulated at the ripening stage, in line with the visual enhancement of red coloration. Interestingly, cuticle- and cell-wall-related genes that showed differential expression between the developmental stages were mainly upregulated in the skin of early fruit, with lower expression at mid-growth and ripening stages. Nevertheless, lignification may be involved in skin hardening in the mature fruit.

## 1. Introduction

Pomegranate (*Punica granatum* L.) fruit is well known for its health-beneficial metabolites [1,2,3,4], particularly anthocyanins and the ellagitannin punicalagin, which accumulate in the arils and peel [5,6]. Due to its medicinal properties, there is high global demand for the fruit. The pomegranate’s appearance affects its marketing and storage. The main concerns related to fruit skin quality are the accumulation of anthocyanins in the peel to obtain the characteristic deep red color and cracking of the fruit surface, which leads to costly yield loss.

The peel of pomegranate fruit consists of an inner thick spongy white tissue (mesocarp/albedo) and an outer smooth skin layer (exocarp/flavedo) which, in red cultivars, turns red when ripe [7]. The fruit skin has a dual role; it protects the fruit from environmental stresses and at the same time plays a critical role in resisting internal growth pressures, controlling fruit expansion, and maintaining fruit integrity. The pomegranate skin is comprised of epidermis cells covered by a cuticle [8]. Symptoms of cracking and russeting in pomegranate develop first as tiny cracks in the cuticle, and cv. Wonderful appears to be more susceptible to this than other cultivars [9]. It has been suggested that cracking is induced by differences in growth rate between the fruit peel and flesh, and the pressure imposed by the quickly expanding arils on the stretched peel [10]. Extreme and changing temperatures may further affect incoming and outgoing water fluxes, adding more strain on the peel [8]. Finding the factors that strengthen the skin against cracking is of significant economic importance.

The biochemical pathway and genetic control of anthocyanin production have been well characterized in numerous plant species and are considered highly conserved among different species in the plant kingdom [11]. In the pomegranate fruit, the dynamics of color development in the skin and arils differs with respect to timing and pigment accumulation during fruit development [2]. While some pomegranate varieties constantly accumulate anthocyanins, others lose their color early and develop it again during the final stages of fruit development [2,12]. During their biosynthesis, anthocyanins and hydrolyzable tannins compete for the same substrate—3-dehydroshikimate—in the shikimate pathway [13]. In addition, several biosynthetic pathways branch off from the main anthocyanin pathway, including those for other flavonoids and isoflavonoids. The metabolic flow and mechanism of regulation of these branched pathways in pomegranate at specific developmental stages are of great importance. Since skin appearance affects the customer’s decision to purchase the fruit, knowledge of the orchestration of these pathways could serve to develop new breeding tools to facilitate color accumulation before harvest.

The developmental transcriptome can shed light on the ripening processes of pomegranate skin and may mirror the respective events occurring in the whole fruit. Due to relatively low respiration rates and low ethylene production, the pomegranate fruit is classified as non-climacteric [2]. In strawberry, the model plant for non-climacteric fruit, abscisic acid (ABA) is the primary plant growth regulator driving fruit ripening [14]. The involvement of ABA or other plant growth regulators in pomegranate ripening is not yet known.

Previous publications have reported on pomegranate genome sequencing and transcriptome analyses [6,15,16,17]; however, these were conducted with the whole peel, including the spongy tissue, focused on the identification and expression of genes involved in anthocyanin and hydrolyzable tannin biosynthesis, and did not emphasize the skin’s ripening processes.

The main pomegranate cultivar in Israel is Wonderful, which blooms in the spring, around the beginning of May, and is harvested toward the end of October. We prepared transcriptomes of pomegranate skin from three developmental stages: early fruit, when the fruit skin is dark green; mid-growth fruit, when the fruit skin is at the color-break stage; and ripening fruit, at harvest. Transcriptome analysis facilitated the monitoring of important characteristics during skin development. We focused on the ripening processes of the skin that relate to its non-climacteric nature, including anthocyanin and hydrolyzable tannin accumulation, and cell wall and cuticle components that may contribute to the skin’s resistance to cracking.

## 2. Materials and Methods

### 2.1. Plant Material

**Figure 1:**
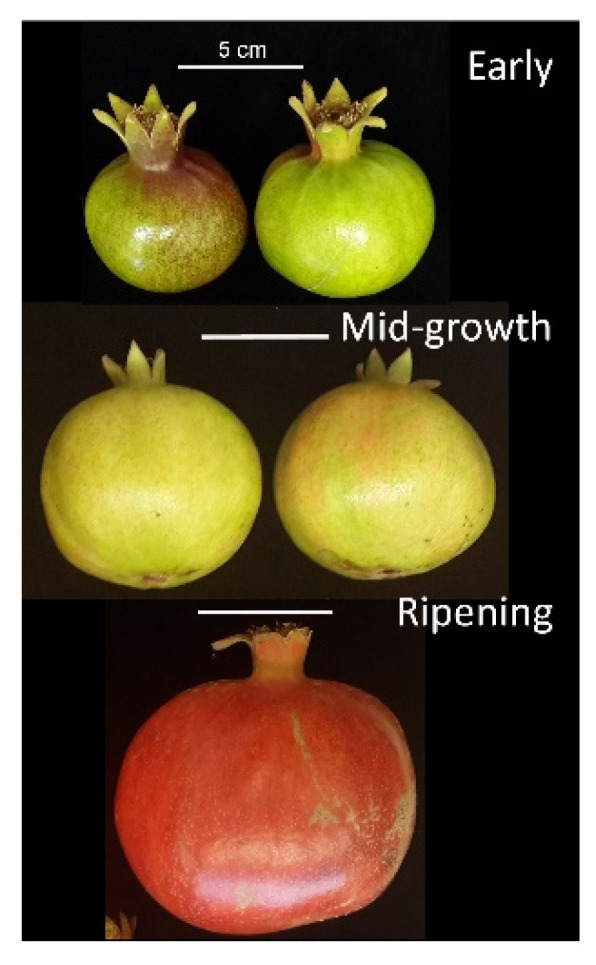
Pomegranate fruit at specified developmental stages. Early, mid-growth, and ripening fruit at 3, 11, and 22 weeks after petal fall, respectively. Bar = 5 cm.

Pomegranate (*Punica granatum* L.) cv. Wonderful fruit were collected from the commercial orchard of Shikma Field Crops, located in Kibbutz Mishmar HaNegev at the northern fringe of the Negev desert (31°22′55.5″ N 34°43′00.8″ E). Fruits were collected by hand at three developmental stages: early fruit, at 3 weeks after petal fall, when the fruit skin is dark green; mid-growth fruit, at 11 weeks after petal fall, when the fruit skin is at the color-break stage; and at ripening, a few days before the beginning of commercial harvest, 22 weeks after petal fall (Figure 1). The commercial harvest lasts for about 3 weeks, during which time the fruit maintains its high quality—firm skin, glossy appearance, and deep red color. The use of pomegranate was carried out in accordance with relevant guidelines and regulations.

The peel of the pomegranate fruit consists of an inner thick spongy white tissue, and an outer thin red skin made up of epidermis cells covered by a cuticle. The transcriptome analyses were performed on the colored skin tissue, which was carefully removed from the spongy tissue, from all sides of the fruit, using a peeler. Skin samples were collected in two biological replicates for each time point, each consisting of three fruits. Skin samples were immediately frozen in liquid nitrogen and kept at −80 °C until use.

### 2.2. RNA-Seq and Bioinformatics Analyses

Total RNA was extracted according to Ginzberg et al. [18]. Purified RNA was sequenced on the Illumina platform, with 100 bp paired-end reads, at the Technion Genome Center, Israel (https://tgc.net.technion.ac.il/). Raw reads were subjected to a filtering and cleaning procedure. The Trimmomatic tool [19] was used to remove Illumina adapters from the reads. Next, the FASTX Toolkit (http://hannonlab.cshl.edu/fastx_toolkit/index.html, version 0.0.13.2) was employed to trim read-end nucleotides with quality scores < 30 using the FASTQ Quality Trimmer, and to remove reads with less than 70% base pairs with a quality score ≤ 30 using the FASTQ Quality Filter. Clean reads were mapped to the reference genome of *P. granatum* (GCF_007655135.1_ASM765513v2) using STAR software (v2.7.1a) [20] with an average mapping rate of 89%. Gene abundance was estimated using Cufflinks (v. 2.2) [21] combined with gene annotations from NCBI. Principal component analysis (PCA) and heatmap visualization were performed using R Bioconductor (accessed on 15 May 2022) [22]. Gene expression values were computed as FPKM. Differential expression analysis was completed using the DESeq2 R package (accessed on 27 April 2021) [23]. Genes with an adjusted *p*-value of no more than 0.05 [24] were differentially expressed. Venn diagrams were performed using the Venn web tool (https://bioinfogp.cnb.csic.es/tools/venny/index.html; accessed on 28 April 2021). Hierarchical cluster analysis of heatmaps was performed with the ClustVis tool (accessed on 28 April 2021) [25]. Note that on the heatmaps, the expression values were normalized for differences between the developmental stages, i.e., the colored tiles represent changes, not absolute values, of gene expression.

The annotation of *P. granatum* proteins was used as a query term for searching the NCBI non-redundant (nr) protein database, which was carried out with the DIAMOND program [26]. The search results were imported into Blast2GO version 4.0 [27] for gene ontology (GO) assignments. Homologous sequences were also identified by searching the Swiss-Prot database with the BLASTx tool (accessed on 27 April 2021) [28] and an *E*-value threshold of 10^−5^, and by comparing to the *Arabidopsis* database (http://www.arabidopsis.org/; accessed on 27 April 2021) and *Malus domestica* proteins (https://data.jgi.doe.gov/refine-download/phytozome?organism=Mdomestica&expanded=491%2CPhytozome-196; accessed on 18 June 2021).

Figures and tables in the manuscript present genes with differential expression between the three developmental stages of the fruit. In addition to the described basic analysis, these genes were annotated manually, and the specific descriptions of their functions were determined based on the activity of their *Arabidopsis* orthologs using the TAIR database and the relevant literature. The KOBAS 3.0 tool (http://kobas.cbi.pku.edu.cn/kobas3/?t=1; accessed on 26 May 2021) was used to detect statistical enrichment of the differentially expressed genes in the KEGG pathway and GO analysis.

## 3. Results and Discussion

### 3.1. Data Validation and Characterization of Pomegranate Skin Developmental Stages

RNA-seq analysis was performed on pomegranate skin collected from fruit at three distinct developmental stages: early fruit with dark green skin, mid-growth fruit at the onset of color break, and ripening fruit at harvest (Figure 1). It should be noted that sampling of the pomegranate skin at earlier than the color-break stage could provide information on genes involved in the initiation of ripening. PC plot and sample correlation matrix were constructed to verify the reproducibility of the biological replicates for each developmental stage of the skin (Figure S1). Skin samples of early, mid-growth, and ripening fruit were significantly different; replicated samples were clustered, although early fruit replicates were slightly separated on the PC plot.

**Figure 2:**
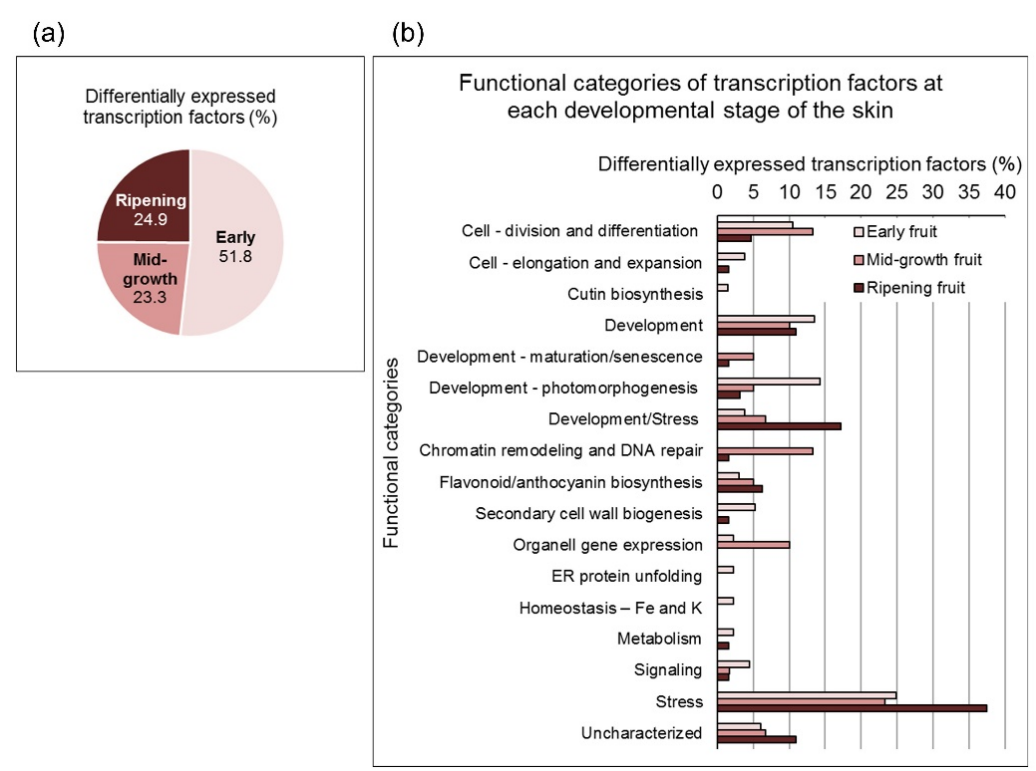
Graphical representation of the percentage of differentially expressed transcription factors in pomegranate skin (**a**) at the three developmental stages, (**b**) categorized into functional groups. The transcript function was determined manually based on the respective *Arabidopsis* orthologs at https://www.arabidopsis.org/.

The transcriptome analysis resulted in 29,281 unique transcripts (Table S1), of which 9835 genes showed differential expression between the three developmental stages of the fruit at *p*_adj_ ≤ 0.05 (Tables S2 and S3). Quantitative reverse transcription PCR (qRT-PCR) confirmed the differential expression of selected genes based on the transcriptome analysis (data not shown). The highest number of differentially expressed genes was found in early compared to ripening fruit (Figure S2a). Accordingly, when the differentially expressed genes were divided into six clusters (Figure S2b and Table S3), most of the differences lay between early and ripening fruit. Using Venn diagrams, it was possible to classify the transcripts that are highly and predominantly expressed in the early and ripening fruit (Figure S3). However, this analysis resulted in hundreds of genes, making it difficult to categorize their function into meaningful biological processes (the KEGG and GO analyses are too general). To better characterize the uniqueness of the skin at each developmental stage, the Venn analysis was used to extract the differentially expressed transcription factors. The specific functions of these regulators reflect the actual biological processes in the tissue. Thus, the sequences of the transcription factors extracted from the Venn analysis were blasted against the TAIR database. Their specific biological function and functional categorization were determined manually according to their respective *Arabidopsis* orthologs (Table S4). More than 50% of the transcription factors were found to be expressed in the skin of early fruit, and the rest were divided almost equally between the skin of mid-growth and ripening fruit (Figure 2a). By categorizing their functions and comparing the skin of early and ripening fruit, we found that the main activities in the early skin were cell division, differentiation and expansion, morphogenesis, secondary cell wall biogenesis and cutin synthesis, ion homeostasis, endoplasmic reticulum activity, and metabolism and signaling—all consistent with early tissue development (Figure 2b). At the same time, ripening skin was characterized by stress response and stress-related developmental processes (Figure 2b). Transcription factors related to flavonoid and anthocyanin biosynthesis were expressed throughout fruit skin development; however, their percentage was higher, as expected, in the skin of ripening fruit (Figure 2b). Some of the activities were missing in the mid-growth stage or showed intermediate values, except for maintenance functions, such as tissue maturation processes, organellar gene expression, and chromatin remodeling and DNA repair, which predominated at this stage.

Interestingly, although pomegranate is a non-climacteric fruit, about 18% of the transcription factors were ethylene-response factors (ERFs). These regulated cell wall biogenesis and signaling in the early fruit skin, and cutin biosynthesis, stress responses, and stress-related developmental processes in early and ripening fruit skin (Table S4). In addition, the transcriptome included two known ripening-related transcription factors: *SHATTERPROOF* (*SHP*) (LOC116194004), which controls ripening in non-climacteric strawberry fruit [29] and climacteric tomato fruit [30] and promotes seed dispersal in dehiscent fruit [31]; and two members of *FRUITFUL* (*FUL*) (LOC116189425, LOC116214111), which influence ripening in an ethylene-independent manner [32]. In fleshy fruit, *SHP* plays an important role in regulating the ripening process in synchronization with seed maturation, transforming the fruit into a seed-dispersal organ [30]. In strawberry, *FaSHP* shows ripening-specific expression and is upregulated by ABA treatment [29]. *FaSHP* downregulation delays anthocyanin accumulation by downregulating *FaCHS* and *FaMYB10* genes, and represses the expression of softening-, aroma-, and ascorbic-acid-related genes [29]. In pomegranate skin, *SHP* and *FUL* expression was found at all three developmental stages with no significant difference.

In analyzing the skin transcriptome, we chose to focus on several topics that characterize pomegranate fruit: (a) its non-climacteric nature, (b) timing of hydrolyzable tannin and anthocyanin biosynthesis, reflecting the accumulation of health-promoting metabolites and visual appearance, and (c) developmental expression of the cell-wall- and cuticle-related genes that potentially affect the resistance of the peel to cracking and thus yield loss.

### 3.2. The Climacteric Nature of Pomegranate Skin

Analyzing the transcriptome of the pomegranate skin provided insights into the non-climacteric nature of the fruit. The mature skin collected at the phenological stage of fruit ripening (22 weeks after petal fall) may mirror the ripening process at the end of fruit growth.

Pomegranate is a non-climacteric fruit. The difference between climacteric and non-climacteric fruit is that the former ripens with concomitant increases in respiration and ethylene production, whereas these barely change in the latter [33]. The fruit-ripening process is associated with color and aroma development, softening, an increase in sugar content, and a decrease in acidity. Most climacteric fruit accumulate starch before the onset of ripening and convert it to soluble sugars at ripening. In contrast, in non-climacteric fruit, the starch content drops rapidly after anthesis, and soluble sugars accumulate throughout development and ripening [34]. This implies that climacteric fruit can be harvested early, and the starch reserve will be converted to sugars during the postharvest stages, whereas non-climacteric fruit should be collected when the desired soluble sugar level is reached, because ripening processes cannot be induced postharvest [34]. In pomegranate skin, genes coding for starch biosynthesis were differentially and highly expressed mainly at the mid-growth phase, and less expressed at the ripening stage, e.g., *ADP GLUCOSE PYROPHOSPHORYLASE* (*AGPase*; LOC116188266), *STARCH SYNTHASE* (*SS*; LOC116207458), and *STARCH BRANCHING ENZYME* (*SBE*; LOC116213578). However, members of the starch breakdown genes were differentially and highly expressed mainly in early skin but also in ripening skin, e.g., *ALPHA-AMYLASE* (*AMY*; LOC116191375 and LOC116212449), and *BETA-AMYLASE* (*BAM*; LOC116208025). Thus, the accumulation of starch and soluble sugars in the skin of the non-climacteric pomegranate requires further clarification.

Tomato and banana are well-studied climacteric fruit, the former serving as a model fruit. Strawberry is a model for non-climacteric fruit, and additional examples are citrus and grape. Whereas in climacteric fruit, ethylene drives the ripening process, in strawberry, the phytohormone ABA promotes ripening [35]. Auxin, cytokinin (CK), gibberellic acid (GA), jasmonic acid (JA), brassinosteroids (BRs), and ethylene have also been suggested to be involved in the development of non-climacteric fruit [14].

#### 3.2.1. ABA

In strawberry and grape, ABA is associated with ripening. In strawberry, ABA content rapidly increases toward the end of growth and during the ripening stages; it is associated with upregulation of a key gene in ABA biosynthesis, 9-CIS-*EPOXYCAROTENOID DIOXYGENASE* (*NCED*), and downregulation of the ABA-deactivation gene *CYP707A4* [35,36,37]. In grape, ABA concentration increases at ripening, resulting in increased expression of anthocyanin biosynthesis genes and anthocyanin accumulation in the skin [38].

**Figure 3:**
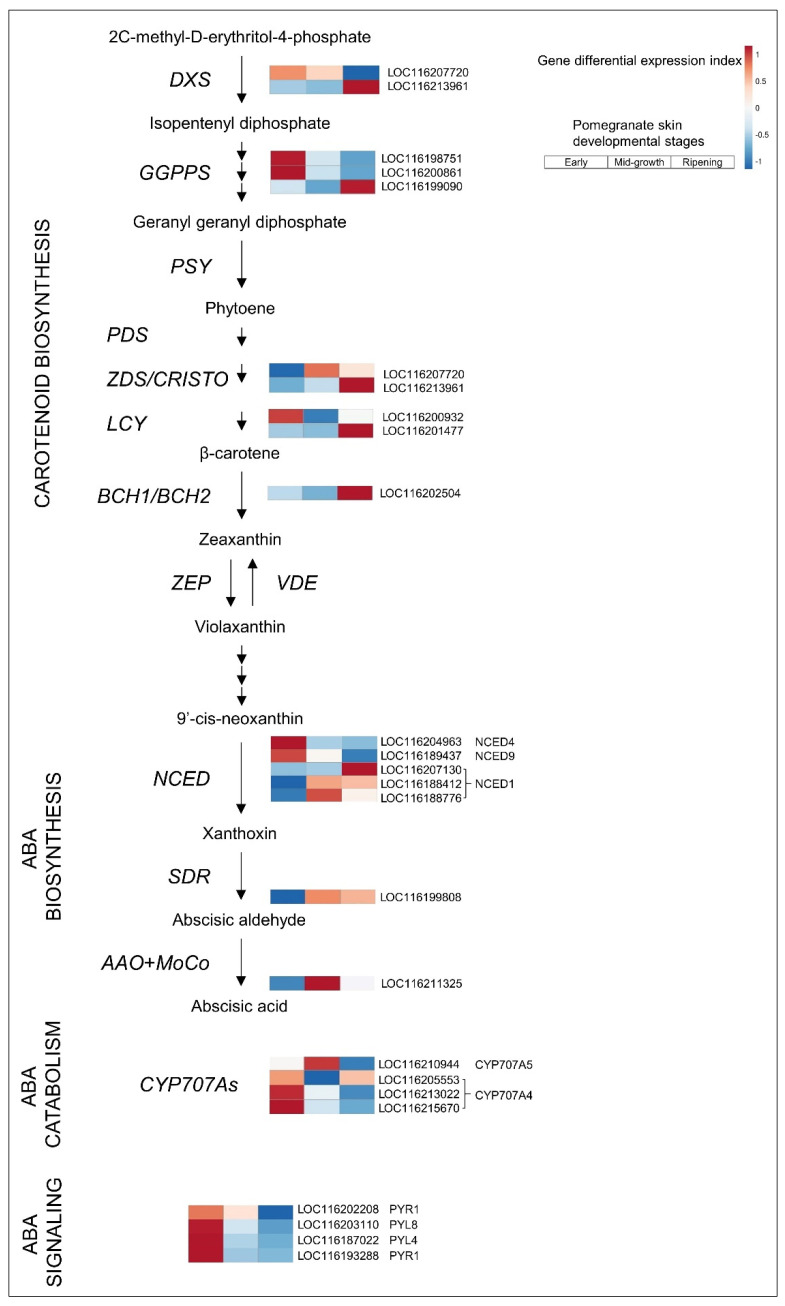
ABA metabolism in pomegranate skin. Illustration of the metabolic pathway combined with heatmap visualization of differentially expressed genes in the skin of early (left column), mid-growth (middle column), and ripening (right column) fruit. Genes involved in each metabolic step are represented by their respective gene IDs. The color index indicates upregulation (red) or downregulation (blue) of a gene relative to the group of genes putatively encoding the same function; expression level values and full gene names are given in Table S5.

In pomegranate, *NCED4* (LOC116204963) and *NCED9* (LOC116189437) were highly and differentially expressed in the skin of early fruit (FPKM 311 and 122, respectively) and strongly downregulated at later stages (FPKM 47 and 45 at ripening, respectively) (Figure 3 and Table S5). The *NCED1* genes (LOC116207130, LOC116188776, and LOC116188412) showed the opposite trend: low expression in early skin and upregulation in mid-growth or ripening skin. However, their highest expression levels (FPKM 1.8, 74 and 18, Table S5) were lower than the levels of *NCED4* and *NCED9* in early skin, suggesting an overall reduction in ABA biosynthesis at later stages of skin ripening. Accordingly, expression upstream of the pathway, where precursors for carotenoid biosynthesis are produced, was mainly found in the ripening skin, implying precursor flow for carotenoid accumulation instead of ABA synthesis (Figure 3 and Table S5).

ABA action is initiated by ABA perception via Regulatory Components of ABA Receptor/Pyrabactin Resistance Protein1/PYR-Like protein (RCAR/PYR1/PYL) that triggers downstream signaling cascades to induce physiological effects [39]. The putative ABA receptor *FaPYR1* acts as a positive regulator in strawberry fruit ripening [40]. Silencing of *FaPYR1* significantly delays strawberry ripening, as indicated by a loss of red coloration. ABA content and ABA sensitivity are also altered, and the transcript level of ABA-responsive gene transcripts is reduced; this cannot be reversed by external application of ABA [40]. In the pomegranate skin transcriptome, there were 10 members of the PYR/PYL family (Table S5); only 4 showed differential expression—higher in early vs. ripening fruit (Figure 3)—including pomegranate *PYR1* (LOC116202208, LOC116193288), an ortholog of *FaPYR1*. Overall, the data are not conclusive with respect to the role of ABA in pomegranate skin ripening. It is suggested that ABA is involved in early fruit development; however, its involvement in initiating early events in the ripening process cannot be ruled out.

#### 3.2.2. Ethylene

**Figure 4:**
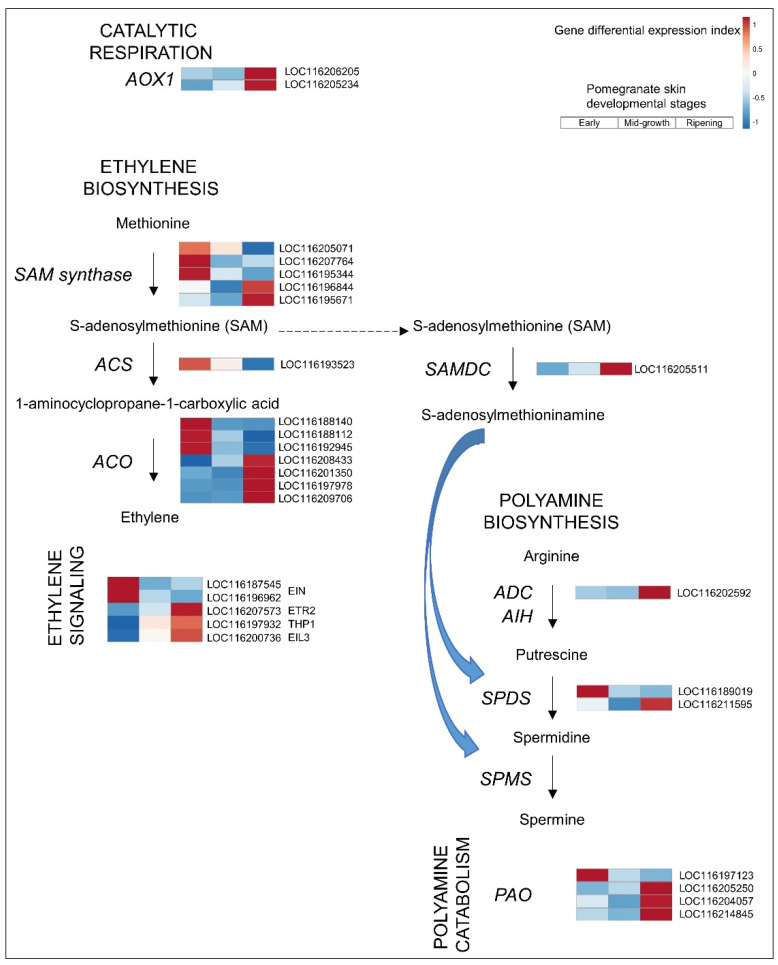
Ethylene and polyamine biosynthesis in pomegranate skin. See Figure 3 legend for details. Expression level values and full gene names are given in Table S6.

As already noted, non-climacteric fruit such as strawberry, grape, raspberry, and citrus are defined by the absence of an ethylene-related respiratory peak and do not show a climacteric rise in ethylene evolution. Pomegranates produce trace amounts of ethylene and show a decline in respiration rate during fruit development [41]. Moreover, postharvest application of ethylene does not significantly affect ripening-related traits such as external color, juice color, total soluble solids, pH, or titratable acidity compared to untreated controls [41]. Although pomegranate is considered a non-climacteric fruit, ethylene plays a role in skin ripening (Figure 4).

Ethylene biosynthesis begins with the formation of S-adenosylmethionine (SAM) from methionine and ATP, catalyzed by SAM synthase. The next two enzymatic steps are catalyzed by 1-aminocyclopropane-1-carboxylic acid synthase (ACS) and 1-aminocyclopropane-1-carboxylic acid oxidase (ACO) to produce the ethylene (Figure 4). SAM can also be diverted to the synthesis of polyamines (PAs), as discussed in Section 3.2.3.

Examination of ethylene biosynthesis in ripening pomegranate skin indicated low expression of *ACS* relative to that in early skin. However, family members of *ACO* were upregulated, with high expression levels (total FPKM 660 and 128 in ripening and early fruit skin, respectively; Table S6) (Figure 4). *ACS* and *ACO* expression have been previously reported in non-climacteric fruit [14]. The expression of ethylene biosynthesis genes and ethylene levels increase in the receptacle of red raspberry during fruit ripening [42]. In strawberry, *ETHYLENE RECEPTOR* (*ETR*) transcript levels increase at the onset of ripening [43]. Similarly, in pomegranate, the *ETR2* transcript (LOC116207573) was differentially upregulated in the ripening skin (Figure 4). *ETR* expression can be found in both climacteric and non-climacteric fruit—the number of *ETR* members is higher in the former, but *ETR* expression occurs earlier in the latter relative to sugar accumulation [44].

Two additional components of the ethylene signaling cascade were upregulated in the ripening skin: THP1, which reduces the ethylene response, and ethylene insensitive 3-like protein (EIL3), related to mineral toxicity stress (Figure 4). As noted in Section 3.1, about 18% of the differentially expressed transcription factors in the pomegranate skin were of the ERF type; a third of these were upregulated in the ripening skin (Table S4). The observed expression of ethylene biosynthesis genes, signaling components, and response factors in the non-climacteric fruit suggests that even the little ethylene produced could activate ripening-related physiological processes [43].

Respiration and ethylene are physiologically correlated during climacteric ripening. A rise in respiration is accompanied by a spike in autocatalytic ethylene production, and their crosstalk has been suggested to be mediated by *ALTERNATIVE OXIDASE* (*AOX*) expression [45,46]. In ripening pomegranate skin, an *AOX* gene was co-upregulated with the *ACO* genes (Figure 4). The data imply that in the non-climacteric pomegranate skin, there is crosstalk between respiration and ethylene metabolism, albeit at a basal level without autocatalytic ethylene production [45].

#### 3.2.3. PAs

PA biosynthesis has also been suggested to have a critical role in fruit ripening [47]. The PAs putrescine (Put), spermidine (Spd), and spermine (Spm) are growth regulators involved in stress response, fruit development, and ripening-like physiological processes. Their biosynthetic pathway starts with arginine decarboxylase (ADC), followed by agmatine iminohydrolase (AIH) of the arginine deiminase family, to produce Put (Figure 4). The conversion of Put to Spd and Spm induces a metabolic transition associated with the onset of fruit ripening [47]. This step is mediated by SAM decarboxylase (SAMDC) activity, which converts SAM, the common substrate for the biosynthesis of PAs and ethylene, to S-adenosylmethioninamine. The latter is used by Spd synthase (SPDS) and Spm synthase (SPMS) to convert Put to Spd and Spm, respectively. The catabolism of Spd and Spm by polyamine oxidase (PAO) further promotes fruit ripening through interaction with other growth regulators, as has been shown for grapes [48].

In the ripening skin of the pomegranate, *ADC*, *SPDS,* and *PAO* were upregulated compared to earlier stages of fruit development (Figure 4 and Table S6), suggesting the involvement of PAs in the pomegranate ripening processes. This is further emphasized by the high expression level of *SAMDC* (LOC116205511, Table S6) in the ripening skin, suggesting diversion of SAM from the ethylene biosynthesis pathway to PA biosynthesis.

In strawberry, Spm is dominant at the onset of fruit coloration and in the ripe fruit [49]. Spm has been suggested to regulate strawberry fruit ripening in an ABA-dominated, IAA-participating, and ethylene-coordinated manner [49]. *SPMS* was not found in the transcriptome of the pomegranate skin, and Spd is assumed to be the dominant PA. Overall, it is suggested that similar to strawberry, the PAs are associated with the ripening of pomegranate fruit skin in coordination with ethylene. SAMDC activity mediates the crosstalk between the two biosynthetic pathways. Unlike strawberry, ABA is not associated with pomegranate skin ripening; however, the phytohormone JA may be involved.

#### 3.2.4. JA

**Figure 5:**
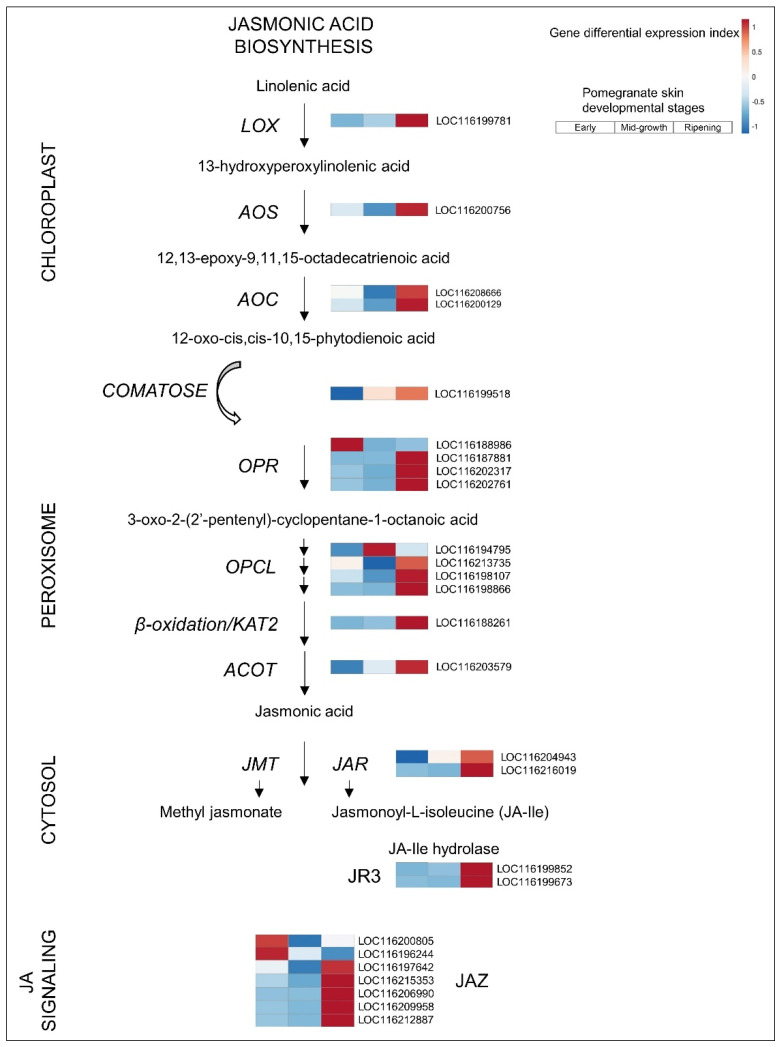
Jasmonic acid biosynthesis and signaling in pomegranate skin. See Figure 3 legend for details. Expression level values and full gene names are given in Table S7.

The entire biosynthetic pathway of JA is upregulated in the ripening skin of pomegranate (Figure 5). This includes upregulation of *JASMONATE RESISTANT 1* (*JAR*), a jasmonic acid–amido synthetase that catalyzes the formation of the biologically active jasmonyl–isoleucine (JA-Ile) conjugate, and is associated with anthocyanin accumulation [50].

In strawberry, the expression of JA biosynthesis genes and related metabolites exhibits significant downregulation concomitant with the increment in ABA levels from the flowering to ripening stages [36,50]. Moreover, exogenous application of methyl jasmonate (MeJA) increases JA, JA-Ile, and MeJA levels, with a concomitant decrease in ABA, suggesting an antagonistic relationship between JA and ABA pathways during ripening of the non-climacteric strawberry fruit [50]. In the ripening pomegranate skin, these trends were reversed—upregulation of JA (Figure 5) and downregulation of ABA (Figure 3) pathways—further distinguishing the ripening process of pomegranate from that of the non-climacteric model. In grape, JA content increases dramatically until the phenological stage of veraison, followed by a sharp decrease during ripening, suggesting that JA is a ripening activator [51].

The biological processes mediated by JA-Ile require activation of the JA signaling pathway. Briefly, once JA-Ile levels are high, the F-box coronatine insensitive1 protein (COI1) ubiquinates the JA signaling repressors jasmonate zim-domain (JAZ)/jasmonate-insensitive 3 (JAI3), activating their degradation by the 26S proteasome [52]. JAZ/JAI3 degradation liberates the MYC2 transcription factor, which then triggers the transcription of JA-responsive genes [52]. In pomegranate, there were 13 members of JAZ/JAI3; 5 of them were upregulated in the skin of ripening fruit (Figure 5 and Table S7), supporting JA signaling activity in ripening.

It is worth noting that JA-Ile is involved in the stress response, and its level needs to be regulated to balance stress and developmental signals [50]. This might involve the stress-responsive jasmonate-responsive 3 (JR3), which regulates the hydrolysis of JA-Ile [53] and is upregulated in the ripening skin (Figure 5 and Table S7). This is particularly relevant to the skin of the ripening pomegranate, where stress responses and stress-related developmental processes account for around 55% of the transcription factor activity (Figure 2 and Table S4).

#### 3.2.5. GA, AUXIN, CK, and BR

The roles of auxin, BR, GA, and CK in the ripening of non-climacteric fruit have not been fully established [14]. In strawberry, indole acetic acid (IAA) and GA_4_ content are high at the phenological stage of fruit enlargement (green stage) and decline as the fruit ripens [36], leading to steep ABA accumulation for fruit ripening [35]. In grape, expression analysis of *BRASSINOSTEROID-6-OXIDASE* (*BR6OX*) and *BRI1-ASSOCIATED RECEPTOR KINASE* indicated upregulation associated with a dramatic increase in endogenous BR level at the onset of fruit ripening, and application of BRs to grape berries significantly promoted ripening [54]. In pomegranate skin, these hormones did not appear to be involved in skin maturation of the ripening fruit. There was no differential expression of most GA biosynthesis genes between skin developmental stages, and GA catabolism occurred in the ripening skin (Figure S4 and Table S8). Genes related to auxin biosynthesis and influx carriers were downregulated in the ripening skin, and active auxin levels were probably further reduced by upregulation of auxin conjugating/hydrolyzing genes (Figure S5 and Table S9). Accordingly, most of the auxin response factors were strongly downregulated in the ripening skin (Figure S5). As for the BRs, *BR6OX* (LOC116206660) and *BRI1* (LOC116207419) expression was low and did not differ between pomegranate skin developmental stages (Table S1).

Taken together, it is suggested that the ripening of pomegranate fruit skin involves the upregulation of ethylene, PA, and JA pathways. This is in line with other studies suggesting that the ripening process is not governed by only one dominant phytohormone, but rather is the result of a controlled balance of several hormones [55].

### 3.3. Upregulated Biosynthetic Pathways of Hydrolyzable Tannins and Anthocyanins

The pomegranate fruit peel contains a high concentration of two groups of polyphenols that confer strong antioxidant activity. The anthocyanins and the hydrolyzable tannins are synthesized from a common intermediate of the shikimate pathway [5,13,56,57].

**Figure 6:**
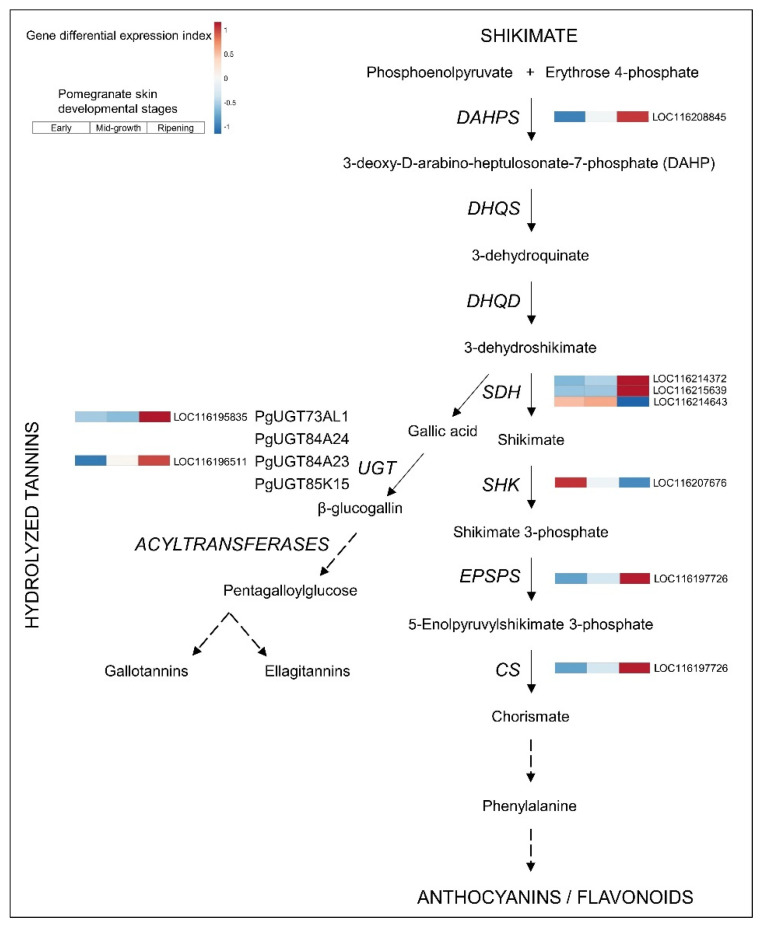
Shikimate and hydrolyzable tannin pathways in pomegranate skin. See Figure 3 legend for details. Expression level values and full gene names are given in Table S10.

The hydrolyzable tannin-specific pathway that biosynthesizes the important ellagitannin punicalagin has not been fully elucidated. It branches from the shikimate pathway, with shikimate dehydrogenase (SDH) as the first committed step catalyzing gallic acid [13] (Figure 6). This is followed by UDP-glucosyltransferase (UGT) activity to produce β-glucogallin, which is converted to pentagalloylglucose by acyltransferase activity [58,59]. Further oxidative modifications of pentagalloylglucose lead to the formation of gallotannins and ellagitannins (e.g., punicalagin in pomegranate) [6,58]. The transcriptome data indicated significant upregulation of two *SDH* (LOC116214372 and LOC116215639) and two of the *UGT* (LOC116195835 and LOC116196511) genes in ripening pomegranate skin (Figure 6 and Table S10), suggesting higher and differential expression in the ripening skin compared to earlier developmental stages of the fruit.

As both hydrolyzable tannin- and anthocyanin-specific biosynthetic pathways divert from the shikimate pathway, it has been argued that they compete for the same precursor metabolites, and thus exhibit opposite trends of accumulation [13]. However, high activity of the related pathways (Figure 6), including the shikimate pathway, may provide sufficient precursors to synthesize both metabolites [13].

**Figure 7:**
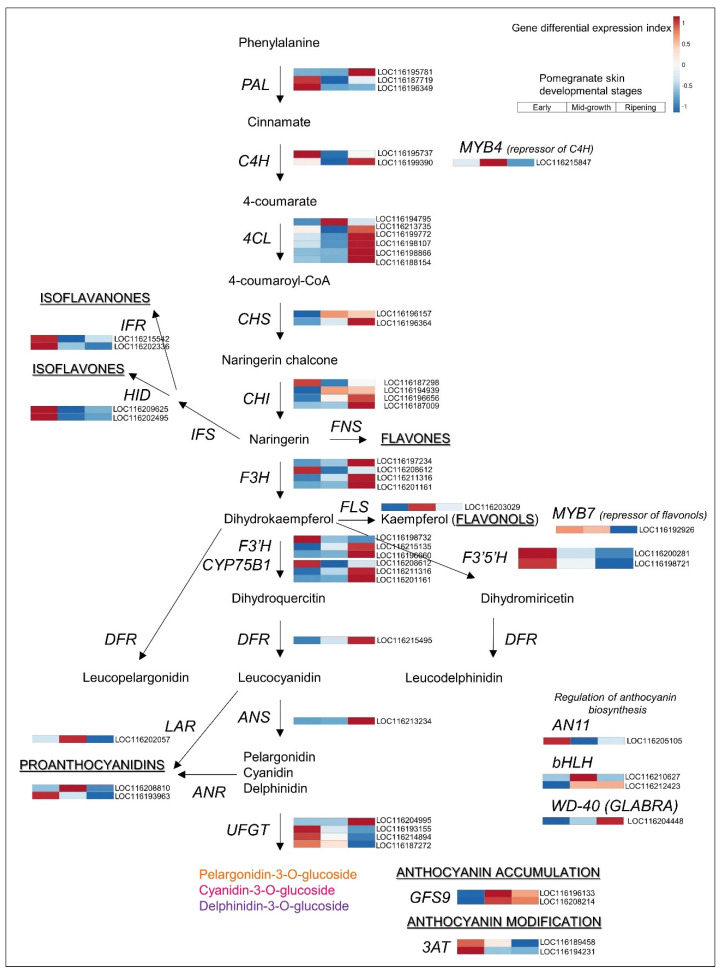
Anthocyanin and flavonoid pathways and regulatory factors in pomegranate skin. See Figure 3 legend for details. Expression level values and full gene names are given in Table S11.

In the well-characterized and conserved anthocyanin biosynthesis pathway [11], common flavonoid precursors are catalyzed by chalcone synthase (CHS), chalcone isomerase (CHI), flavanone 3-hydroxylase, dihydroflavonol 4-reductase (DFR), and leucoanthocyanidin dioxygenase. The expression of *DFR* is correlated with pomegranate color and the timing of color appearance during fruit development [12]. In pomegranate skin, the entire anthocyanin-specific biosynthetic pathway, and its regulator *GLABRA* (WD-40, LOC116204448) [12], and the upstream segment of the phenylpropanoid pathway, including *CINNAMATE 4-HYDROXYLASE* (*C4H*, LOC116199390) and *4-COUMARATE:COA LIGASE* (*4CL*, LOC116199772 and LOC116213735), were significantly and highly upregulated, with high expression values, in the ripening skin (Figure 7 and Table S11). Glucosylation of the anthocyanidins by *UDP-GLUCOSE:FLAVONOID 3-O-GLUCOSYLTRANSFERASE* (*UFGT*) was mainly downregulated in the ripening skin except LOC116204995 that was upregulated; the resulted total expression level was probably sufficient to produce the mono- and diglucosides of cyanidin (red pigments), delphinidin (purple pigments), and pelargonidin (orange pigments) (Table S11) [60,61,62,63]. In pomegranate cv. Wonderful skin, delphinidin glucoside is the major metabolite, followed by cyanidin and pelargonidin glucosides [64].

It should be noted that the trends in expression of the biosynthesis genes of the isoflavanones, isoflavones, flavonols, and proanthocyanidin pathways were opposite that of the anthocyanins—high expression in the skin of early fruit and strong downregulation in the ripening skin (Figure 7 and Table S11)—further supporting the channeling of precursors to anthocyanin biosynthesis during fruit skin ripening.

### 3.4. Strengthening Pomegranate Skin against Cracking: Cutin, Suberin, Wax, and Cell Wall

Pomegranate peel is prone to cracking, which is probably induced by differences in growth rate between the fruit peel and flesh, and the pressure imposed by the quickly expanding arils on the stretched peel [10]. Extreme and changing temperatures may further affect incoming and outgoing water fluxes, adding more strain on the peel [8]. In addition, the anatomy of the skin and peel characteristics may affect skin susceptibility to cracking and fruit splitting.

An anatomical study showed that the skin of ripening pomegranate fruit is made up of epidermal cells that are relatively flat and spaced apart; these were expected to be less durable under internal pressure [8]. In contrast, the skin of early immature fruit had two layers of dense and rounded epidermis, which were expected to be more resistant to cracking. Tensile strength studies confirmed this trend: the skin of mature fruit had a lower elastic modulus than that of young fruit [8].

**Figure 8:**
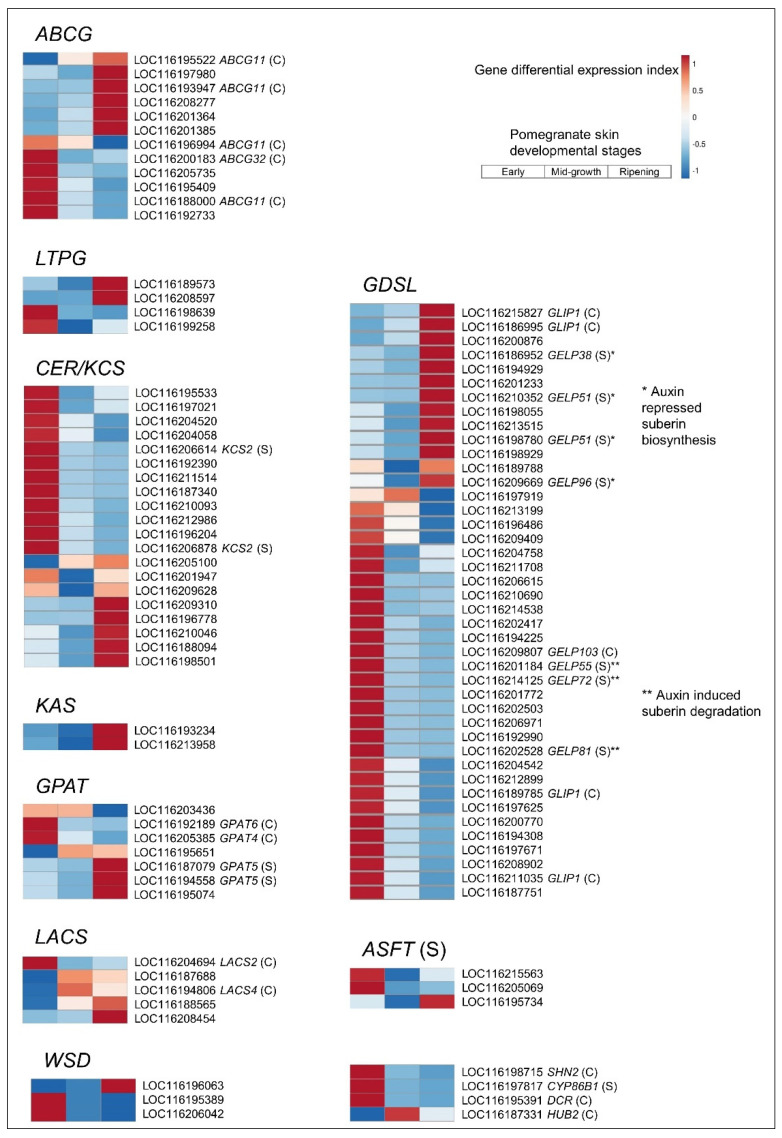
Cutin and suberin biosynthesis genes in pomegranate skin. See Figure 3 legend for details. *Arabidopsis* orthologs’ gene codes are indicated next to the pomegranate gene ID; (C) and (S) indicate verified involvement in cutin and suberin biosynthesis, respectively, based on [65,66]. Expression level values and full gene names are given in Table S12.

Symptoms of cracking and russeting in pomegranate initiate as tiny cracks in the cuticle, and cv. Wonderful appears to be more susceptible to this than other cultivars [9]. This could be due to the different timing of its fruit set and harvest—Wonderful is a late cultivar. Nevertheless, because cracking appears first at the cuticle level, cuticle-related genes were identified in the skin transcriptome, and their differential expression during fruit development was determined by heatmap analysis. These genes included the *ABCG* transporters, *NON-SPECIFIC LIPID-TRANSFER PROTEIN* (*LTPG*), *3-KETOACYL-COA SYNTHASE* (*KCS*), β-*KETOACYL–ACYL CARRIER PROTEIN SYNTHASE* (*KAS*), *GLYCEROL-3-PHOSPHATE 2-O-ACYLTRANSFERASE* (*GPAT*), *LONG-CHAIN ACYL-COA SYNTHETASE* (*LACS*), *WAX SYNTHETASE* (*WSD*), *GDSL ESTERASE/LIPASE*, and *ALIPHATIC SUBERIN FERULOYL TRANSFERASE* (*ASFT*) (Figure 8). Orthologs of these genes in the *Arabidopsis* genome were identified to verify their role in cutin or suberin biosynthesis (Figure 8; TAIR gene code is mentioned next to the relevant transcripts) [65,66]. Suberin is a macromolecule composed of cutin-like and lignin-like domains [67], forming the russeting tissue that develops on fruit skin at microcracking sites. It also appears as fine netting on the surface of the ripened pomegranate upon skin senescence.

Heatmap analysis indicated that most of the *KCS* and *GDSL* members were differentially and more highly expressed in the skin of early fruit compared to ripening fruit, whereas for other gene families, around half of the members were upregulated in the skin of early fruit and half in the ripening skin (Figure 8 and Table S12). In general, no differential expression was detected for the skin of mid-growth fruit. Unexpectedly, suberin-related *Arabidopsis* orthologs of pomegranate were not upregulated exclusively in the ripening skin (Figure 8).

Most of the cell-wall-related genes were upregulated in the early fruit compared to ripening fruit; these included *EXPANSIN*, *POLYGALACTURONASE*, *β-GALACTOSIDASE*, *XYLOGLUCAN ENDOTRANSGLUCOSYLASE/ HYDROLASE*, and *PECTIN METHYLESTERASE* (Figure S6 and Table S13). Members of *CELLULOSE SYNTHASE* were upregulated differentially at all three developmental stages.

Heatmap analyses of key steps in lignin biosynthesis indicated differential expression of individual family members at all three developmental stages (Figure S6). However, the expression levels (FPKM values) of the individual genes and the total expression level of the whole gene family were higher in ripening skin than in earlier developmental stages (Table S14). Overall, it is suggested that the skin of ripening pomegranate fruit is hardened by lignification. The lignin can fill the space between the cell wall polysaccharides and increase the cell wall’s mechanical strength [68]. However, high lignification may limit the extensibility of the cell wall, enhancing fruit cracking [69].

## 4. Conclusions

Skin transcriptome analyses suggest involvement of the combined action of ethylene (albeit in trace amounts), PAs, and JA in pomegranate skin ripening. This differs from the ripening mechanism of the model non-climacteric strawberry fruit. However, ABA involvement in initiating early events in the ripening process cannot be ruled out. The pathways of hydrolyzable tannins and anthocyanins are co-upregulated in the skin during ripening, despite competing for the same precursors of the shikimate pathway. The respective genes are downregulated in the skin of early fruit, where flavonoid biosynthesis predominates. Hardening of the fruit peel is suggested to occur by lignification. The data found for the skin may mirror processes occurring at the whole fruit level, warranting further elucidation. The data may provide new ideas for the regulation of pomegranate ripening pre- and postharvest.

## Supplementary Materials

The following are available online at https://www.mdpi.com/article/10.3390/cells11142215/s1. Figure S1. Quality validation; Figure S2. Number of transcripts showing differential expression; Figure S3. Venn analysis of differential transcripts; Figure S4. Gibberellic acid biosynthesis and signaling in pomegranate skin; Figure S5. Auxin metabolism and regulation in pomegranate skin; Figure S6. Cell-wall-related genes in pomegranate skin; Table S1. Transcriptome FPKM; Table S2. DEG analysis; Table S3. List of all differentially expressed genes; Table S4. List of differentially expressed transcription factors; Table S5, list of genes involved in ABA metabolic pathway and signaling, Table S6, list of genes involved in ethylene and polyamines metabolic pathways and signaling, Table S7, list of genes involved in jasmonic acid metabolic pathway and signaling, Table S8, list of genes involved in gibberellic acids metabolic pathway and signaling, Table S9, list of genes involved in auxin metabolism and regulation, Table S10, list of genes involved in hydrolyzed tanins metabolic pathway, Table S11, list of genes involved in anthocyanins and flavonoids metabolic pathway and regulation, Table S12, list of genes involved in cutin and suberin biosynthesis, Table S13, list of genes involved in cell wall biosynthesis, Table S14, list of genes involved in lignin biosynthesis.
