# Supplementary material for: Ripening of Pomegranate Skin as Revealed by Developmental Transcriptomics
Source: Cells. 2022 Jul 16;11(14):2215. doi: 10.3390/cells11142215 (PMC9320897; doi:10.3390/cells11142215)
Supplement: Supplementary file 1 [file cells-11-02215-s001.zip › Figures S1-S6_R3.pdf]

(a)

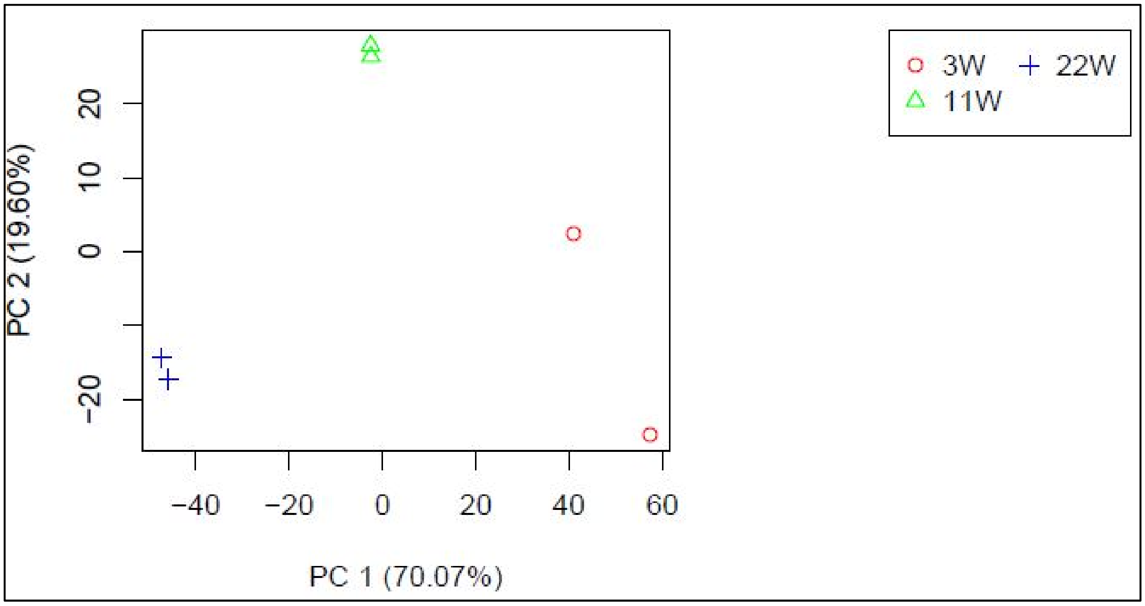

(b)

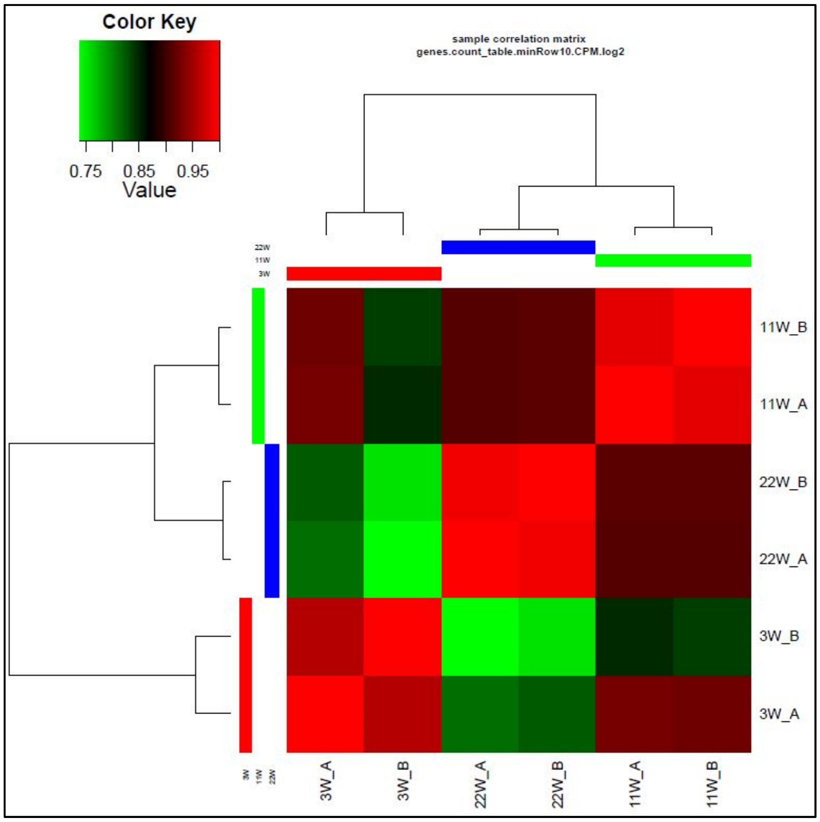

Figure S1. Quality validation. PC plot (a) and sample correlation matrix (b) showing the reproducibility of the biological replicates for each developmental stage of the skin: early, mid-growth and ripening fruit (3, 11 and 22 weeks after petal fall, respectively)

(a)

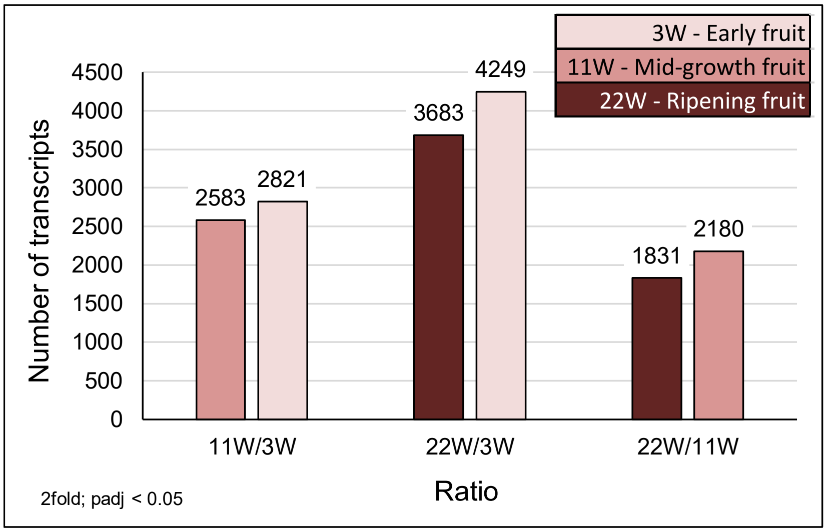

(b)

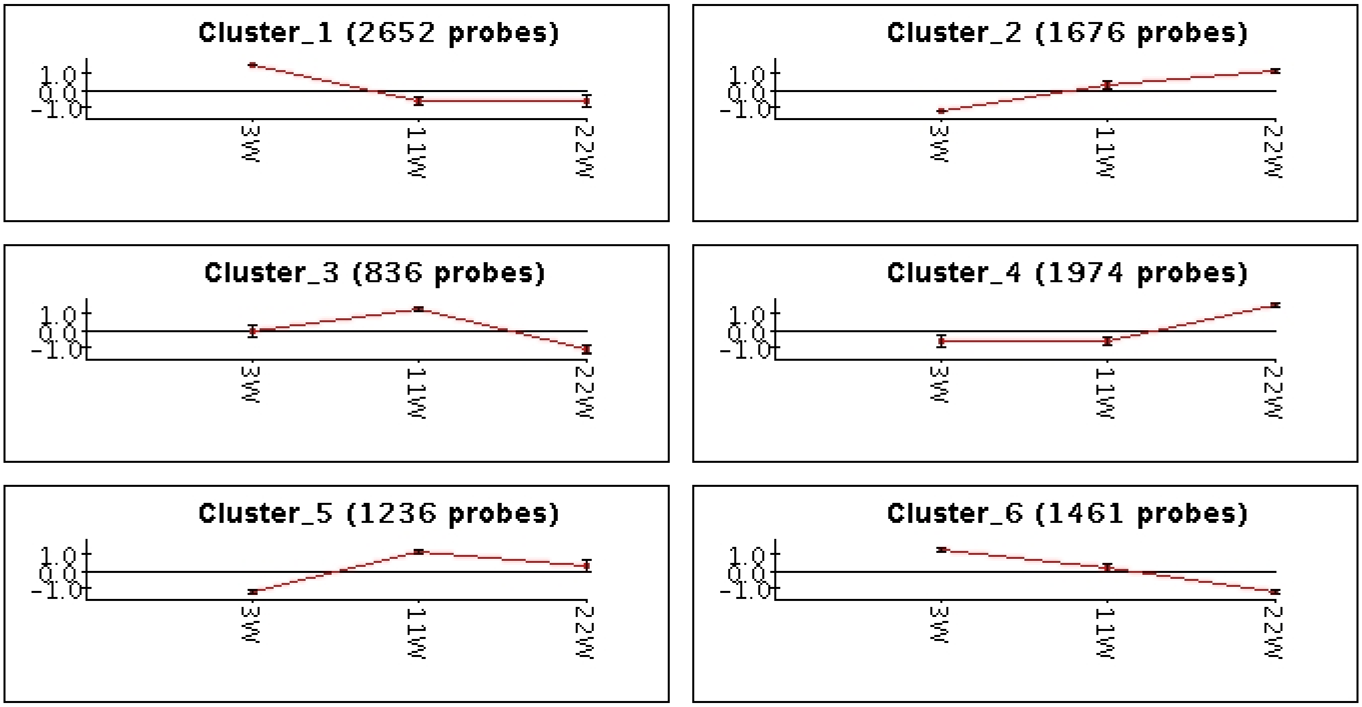

Figure S2. Number of transcripts showing differential expression. (a) Bars represent the number of differentially expressed genes, as an outcome of the comparison between any two developmental stages of the skin: early, mid-growth and ripening fruit (3, 11 and 22 weeks after petal fall, respectively). (b) Cluster analysis demonstrating the expression profiles of the differentially expressed genes. List of genes and their clustering is given in Table S3

Differentially high mainly in early fruit

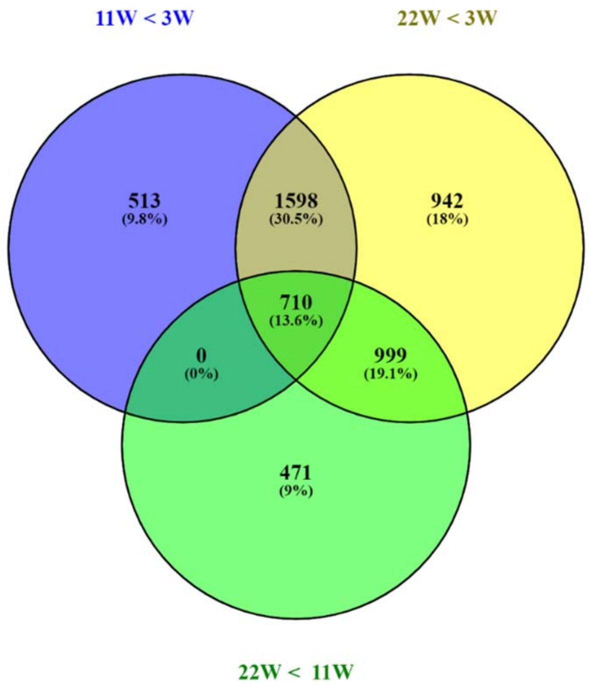

Differentially high mainly in ripening fruit

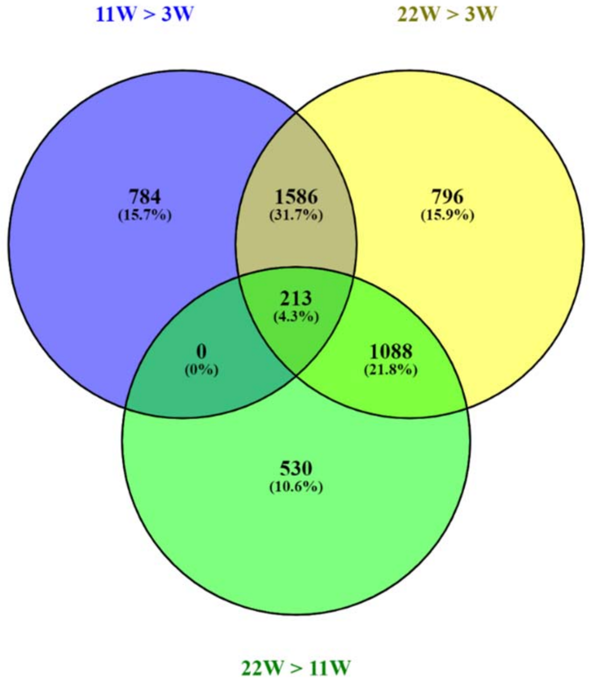

Figure S3. Venn analysis of differential transcripts. Venn diagrams comparing transcripts with differential expression in early, mid-growth and ripening fruit skin (3, 11 and 22 weeks after petal fall, respectively)

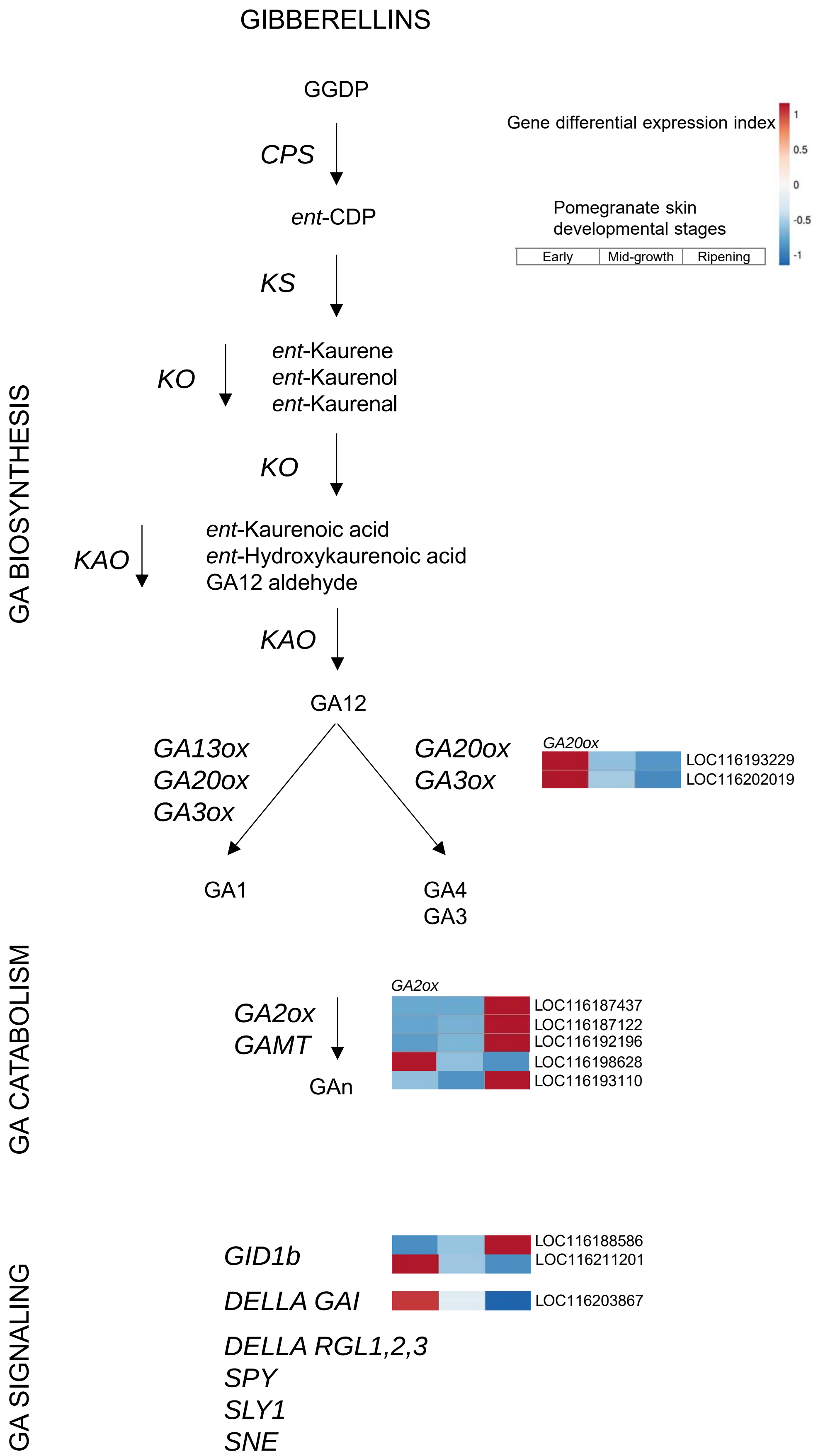

Figure S4. Gibberellic acid biosynthesis and signaling in pomegranate skin. See Figure 3 legend in the main text for details. Expression level values and full gene names are given in Table S8

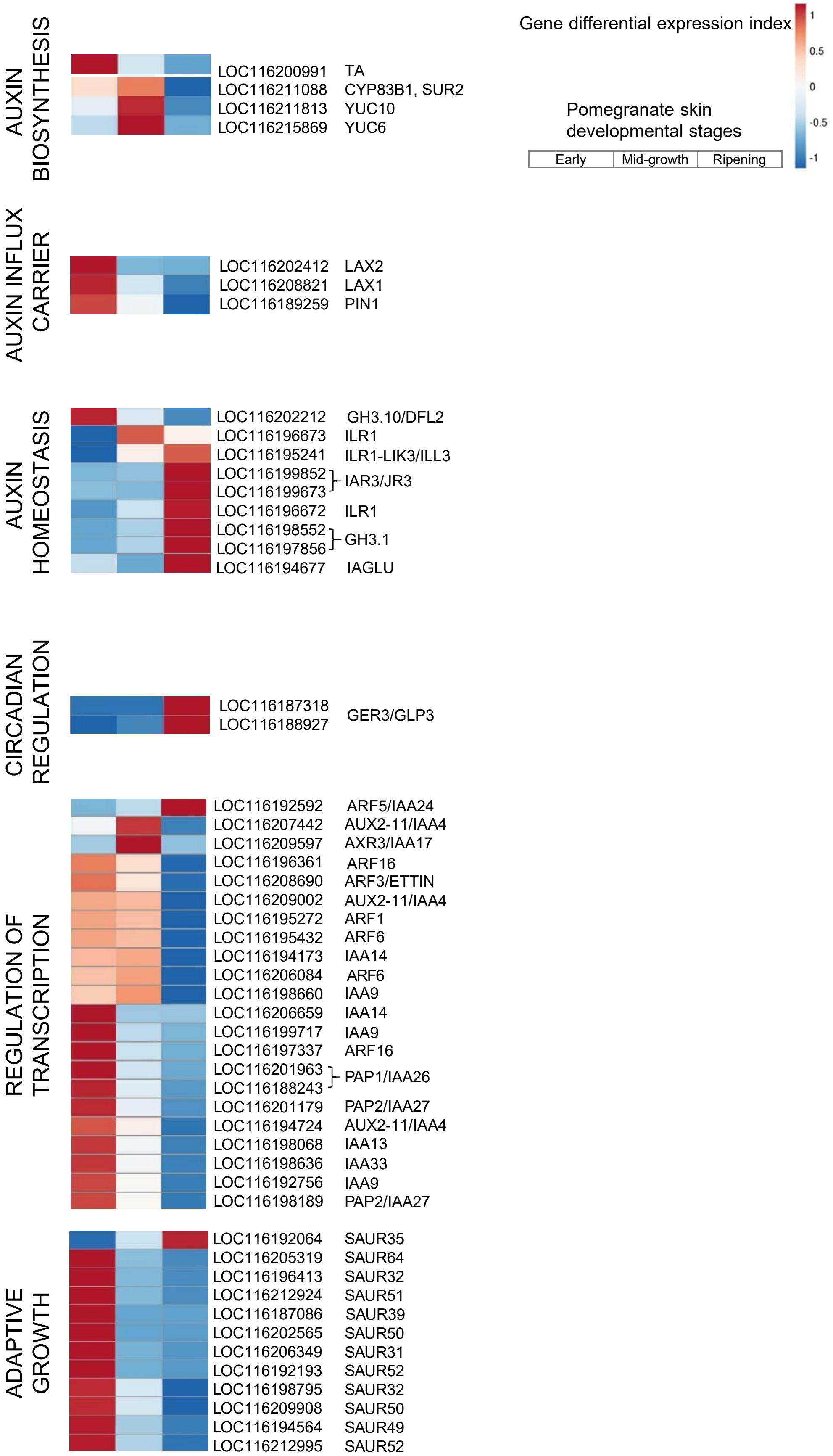

Figure S5. Auxin metabolism and regulation in pomegranate skin. See Figure 3 legend in the main text for details. Expression level values and full gene names are given in Table S9

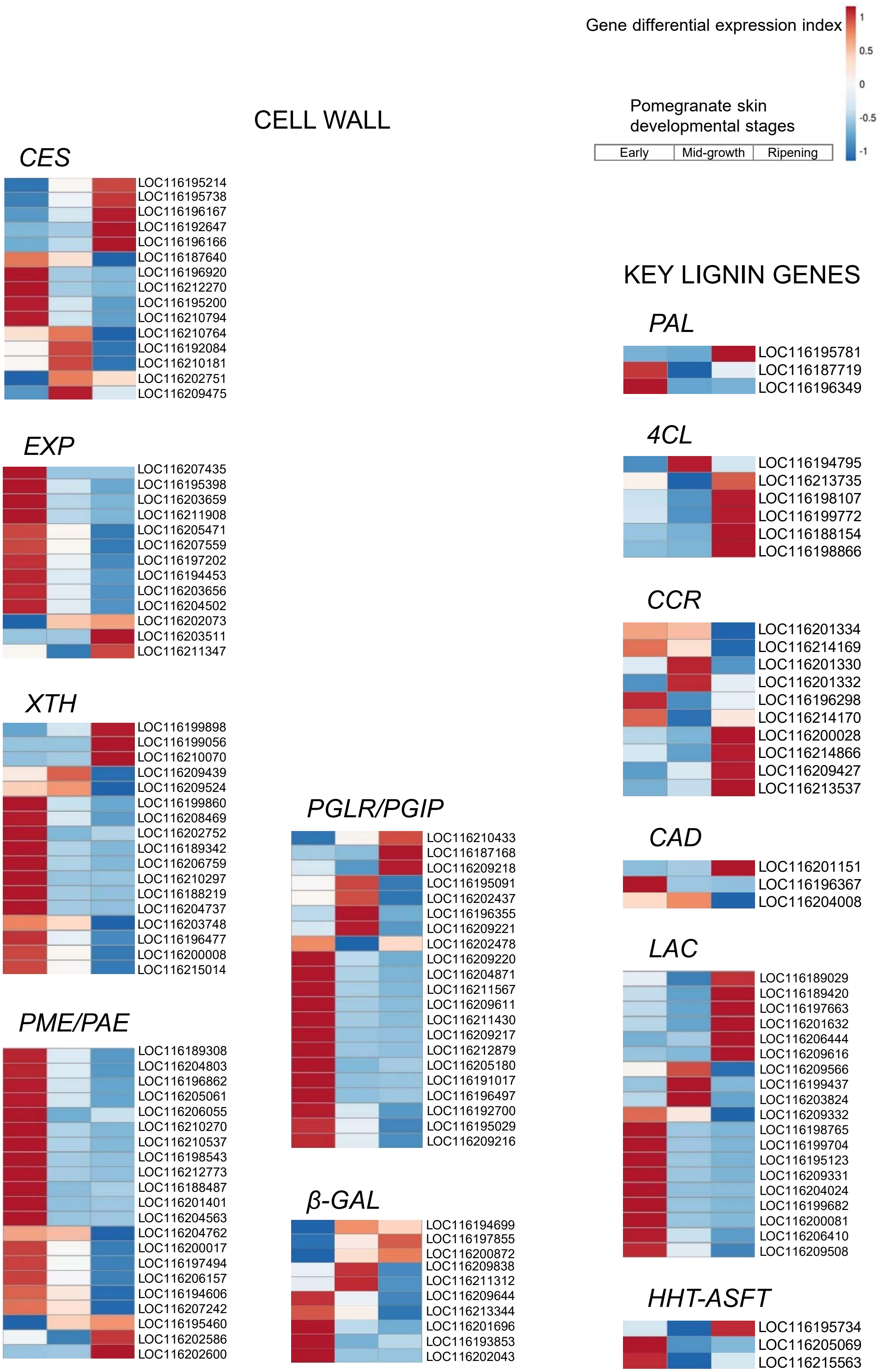

Figure S6. Cell wall-related genes in pomegranate skin. See Figure 3 legend in the main text for details. Expression level values and full gene names are given in Tables S13 and S14
